# Tailored for Real-World: A Whole Slide Image Classification System Validated on Uncurated Multi-Site Data Emulating the Prospective Pathology Workload

**DOI:** 10.1038/s41598-020-59985-2

**Published:** 2020-02-21

**Authors:** Julianna D. Ianni, Rajath E. Soans, Sivaramakrishnan Sankarapandian, Ramachandra Vikas Chamarthi, Devi Ayyagari, Thomas G. Olsen, Michael J. Bonham, Coleman C. Stavish, Kiran Motaparthi, Clay J. Cockerell, Theresa A. Feeser, Jason B. Lee

**Affiliations:** 1 Proscia Inc., Philadelphia, Pennsylvania USA; 2 Department of Dermatology, Boonshoft School of Medicine, Wright State University School of Medicine, Dayton, Ohio USA; 3 Dermatopathology Laboratory of Central States, Dayton, Ohio USA; 4 Department of Dermatology, University of Florida College of Medicine, Gainesville, Florida USA; 5 Cockerell Dermatopathology, Dallas, Texas USA; 6 Departments of Dermatology and Cutaneous Biology, Sidney Kimmel Medical College at Thomas Jefferson University, Philadelphia, Pennsylvania USA

**Keywords:** Classification and taxonomy, Computational models, Machine learning

## Abstract

Standard of care diagnostic procedure for suspected skin cancer is microscopic examination of hematoxylin & eosin stained tissue by a pathologist. Areas of high inter-pathologist discordance and rising biopsy rates necessitate higher efficiency and diagnostic reproducibility. We present and validate a deep learning system which classifies digitized dermatopathology slides into 4 categories. The system is developed using 5,070 images from a single lab, and tested on an uncurated set of 13,537 images from 3 test labs, using whole slide scanners manufactured by 3 different vendors. The system’s use of deep-learning-based confidence scoring as a criterion to consider the result as accurate yields an accuracy of up to 98%, and makes it adoptable in a real-world setting. Without confidence scoring, the system achieved an accuracy of 78%. We anticipate that our deep learning system will serve as a foundation enabling faster diagnosis of skin cancer, identification of cases for specialist review, and targeted diagnostic classifications.

## Introduction

Every year in the United States, 12 million skin lesions are biopsied^1^, with over 5 million new skin cancer cases diagnosed^2^. After a skin lesion is biopsied, the tissue is fixed, embedded, sectioned, and stained with hematoxylin and eosin (H&E) on glass slides, ultimately to be examined under microscope by a dermatologist, general pathologist or dermatopathologist who provides a diagnosis for each tissue specimen. Owing to the large variety of over 500 distinct skin pathologies^3^ and the severe consequences of a critical misdiagnosis^4^, diagnosis in dermatopathology demands specialized training and education. Although the inter-observer concordance rate in dermatopathology is estimated to be between 90 and 95%^5,6^, there are some distinctions which present frequent disagreement among pathologists, such as in the case of melanoma vs. melanocytic nevi^7–11^. Any system which could improve diagnostic accuracy provides obvious benefits for dermatopathology labs and patients; however, there are substantial benefits also to improving the distribution of pathologists’ workloads^12–14^. This can reduce diagnostic turnaround times in several scenarios. For example, when skin biopsies are interpreted initially by a dermatologist or a general pathologist, prior to referral to a dermatopathologist, it can result in a delay of days, sometimes in critical cases. In another common scenario, additional staining is required to identify characteristics of the tissue not captured by standard H&E staining. If those additional stains are not ordered early enough, there can be further delays to diagnosis. An intelligent system to distribute pathology workloads could alleviate some of these bottlenecks in lab workflows. The rise in adoption of digital pathology^1,15^ provides an opportunity for the use of deep learning-based methods for closing these gaps in diagnostic reliability and efficiency^16,17^.

In recent years, deep neural networks have proven capable of identifying diagnostically relevant patterns in radiology and pathology^18–25^. While deep learning applied to medical imaging-based diagnostic applications has progressed beyond proof-of-concept^18,20,22–24^, the translation of these methods to digital pathology must overcome unique challenges. Among these is sheer image size; a typical whole slide image (WSI) scanned at 20x objective power contains anywhere from several hundred megabytes to 1 gigabyte of data and billions of pixels. Additionally, non-standardized image appearance (variability in tissue preparation, staining, scanned appearance, presence of artifacts) and a large number of pathologic abnormalities that can be observed present unique barriers to development of deployable deep learning applications in pathology. For example, Tellez *et al*.^26^ demonstrate the strong impact that inter-site variance– with respect to stain and other image properties– can have on deep learning models. Nonetheless, deep learning-based methods have recently shown promise in a number of tasks in digital pathology, primarily for segmentation models and networks which classify small patches within a WSI^19,26–35^. More recent methods have performed direct WSI classification^21,25,36^, with most applications primarily focusing on making binary classifications^19,25,31,36^. There has been less focus on applications which demand classification of multiple pathologies of interest. Additionally, many of these methods have focused on curated datasets consisting of fewer than 5 pathologies with little diagnostic and image variability^19,21,31,36^.

The insufficiency of models developed and tested using small curated datasets such as CAMELYON^29^ was effectively demonstrated by Campanella *et al*.^25^ In contrast to deep learning systems exposed to contrived pathology problems and datasets, pathologists are trained to recognize hundreds of morphological variants of diseases they are likely to encounter in their careers and must adapt to variations in tissue preparation and staining protocols. In addition to these variations, deep learning algorithms can also be sensitive to image artifacts. Some research has attempted to account for these issues by detecting and pre-screening image artifacts, either by automatically^37–39^ or manually removing slides with artifacts^19,25,31^. The recent work of Campanella *et al*. made substantial progress towards validating a deep learning system on data completely free of curation^25^. Their work presented a system trained to detect prostate cancer and underlined the importance that a “clinical-grade” system be trained on a large set of data inclusive of many types of artifacts. However, the study did not demonstrate robust performance in the presence of several types of biological and image variability such a system is expected to encounter in clinical practice. In terms of biological variability, the study does not demonstrate performance on a common diagnostic entity–high grade prostatic intraepithelial neoplasia (HGPIN). However, HGPIN represents 4–16% of prostate needle biopsies and can mimic the appearance of prostate cancer^40–42^, and therefore could be expected to reduce the specificity of such a system. The work of Campanella *et al*. additionally was unprecedented in terms of image variability covered–it includes a large dataset encompassing slides prepped in 44 countries, representing a degree of variability in staining and tissue preparation characteristics which previous studies have lacked. However, the system’s performance suffered due to this variability: a 14% false positive rate was shown in this external set compared to 5% in slides stained and prepped in-house. Campanella *et al*. additionally selectively exclude slides with ink markings, which have been shown to affect predictions of neural networks^43^. However, *inclusion* of slides with pen ink during training of such a system could also cause unexpected performance. For prostate biopsies in particular, pen ink is used to delineate areas indicative of malignancy^21,44^, and thus presence of pen ink can be highly correlated with presence of cancer. Such seemingly innocuous spurious correlates of pathology represent hidden stratification in medical image datasets and if not properly handled in the training of clinical systems, ultimately pose potential for patient harm as Oakden-Rayner *et al*. recently demonstrated^45^. Therefore, we propose that uncurated data, while important, is not a stand-alone requirement for developing and validating a real-world pathology system, and must be paired with careful and comprehensive coverage of the range of biological and image variability on which the system is expected to reliably perform. A real-world deep learning pathology system must be demonstrably robust to these variations. It must be tested on non-selected specimens, with no exclusions and no manual pre-screening of slides input or post-screening of the system outputs. A comprehensive test set for robustly assessing system performance should contain images:

1. From multiple labs, with markedly varied stain and image appearance due to imaging using different whole slide image scanner models and vendors, and variability in tissue preparation and staining protocols.
2. Wholly representative of a diagnostic workload in the subspecialty (i.e. not excluding pathologic or morphologic variations which occur in a sampled time-period).
3. With a host of naturally-occurring and human-induced artifacts: scratches, tissue fixation artifacts, air bubbles, dust and dirt, smudges, out-of-focus or blurred regions, scanner-induced misregistrations, striping, pen ink or letters on slides, inked tissue margins, patching errors, noise, color/calibration/light variations, knife-edge artifacts, tissue folds, and lack of tissue present.
4. With no visible pathology (in some instances), or with no conclusive diagnosis, covering the breadth of cases occurring in diagnostic practice.

In this work, we present a pathology deep learning system (PDLS) which is capable of classifying WSIs containing H&E-stained skin biopsies or excisions into diagnostically-relevant classes (Basaloid, Squamous, Melanocytic and Other). A key aspect of our system is that it returns a measure of confidence in its assessment; this is necessary in such classifications because of the wide range of variability in the images. A real-world system should not only return accurate predictions for commonly occurring diagnostic entities and image appearances, but also flag the non-negligible remainder of images whose unusual features lie outside the range allowing reliable model prediction. The PDLS is developed using 5,070 WSIs from a single lab (*“Reference Lab”*), and independently tested on a completely uncurated and unrefined set of 13,537 sequentially accessioned H&E-stained images from 3 additional labs, each using a different scanner and different staining and preparation protocol. No images were excluded. To our knowledge, this test set is the largest in pathology to date. Our PDLS satisfies all the criteria listed above for real-world assessment, and is therefore to our knowledge the first truly real-world-validated deep learning system in pathology.

## Results

### Overview and evaluation of PDLS

**Figure 1:**
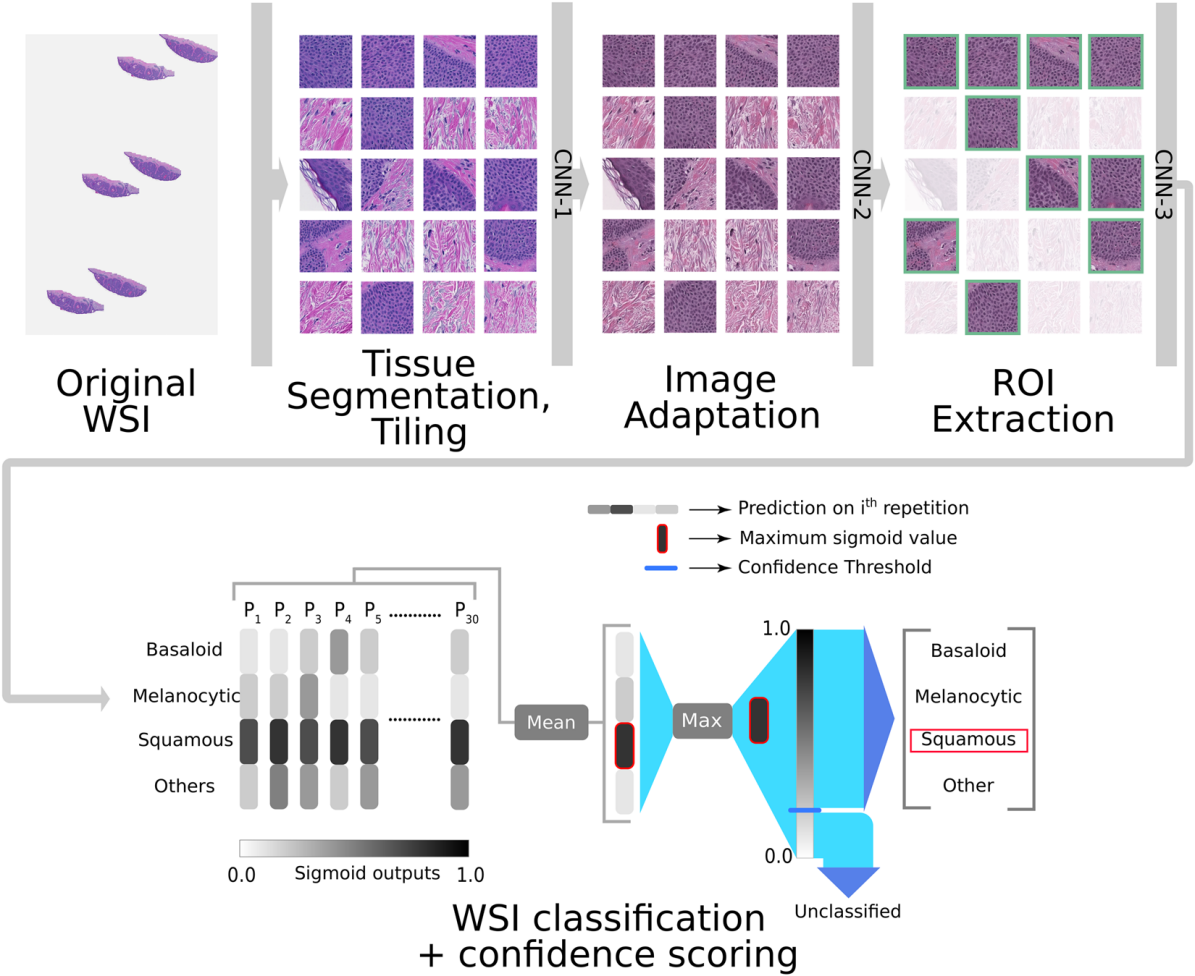
The process of classifying a whole slide image (WSI) with the pathology deep learning system is shown. The input WSI is first segmented and divided into tissue patches (Tissue Segmentation, Tiling); those patches pass through CNN-1, which adapts their stain and appearance to the target domain; they then pass through CNN-2 which identifies the regions of interest (patches) required to pass to CNN-3, which performs a 4-way classification, and repeats this 30 times to yield 30 predictions, where each prediction *P*_*i*_ is a vector of dimension *N*_*classes*_ = 4; the max of the class-wise mean of sigmoid output is the confidence score. If the confidence score surpasses a single class-independent pre-defined threshold set by performance in the Reference Lab, (see Methods) the corresponding class decision is assigned.

The proposed system, as illustrated in Fig. 1, takes as input a WSI and classifies it using a cascade of three independently-trained convolutional neural networks (CNNs) as follows: The first (*CNN-1*) adapts the image appearance to a common feature domain, accounting for variations in stain and appearance; the second (*CNN-2*) identifies regions of interest (ROI) for processing by the final network (*CNN-3*), which classifies the WSI into one of 4 classes defined broadly by their histologic characteristics—Basaloid, Melanocytic, Squamous, and Other, as further described in Methods. Although the classifier operates at the level of an individual WSI, some specimens are represented by multiple WSIs, and therefore these predictions are aggregated to produce a single specimen-level classification. The classifier is trained such that for each image a predicted class is returned along with a confidence in the accuracy of the outcome. This allows discarding of predictions that are determined by the PDLS as likely to be false. Since there is a large amount of variation in both pathologic findings of skin lesions as well as scanner or preparation-induced abnormalities, it is very important for the model to assess a confidence score for each decision; thereby, likely-misclassified images can be flagged as such. We developed a method of confidence scoring based on Gal *et al*.^46^ and set confidence thresholds *a priori* based only on performance on the validation set of the Reference Lab, which is independent of the data for which we report all measures of system performance (see Methods). Three confidence thresholds were calculated and fixed based on the Reference Lab validation set such that discarding specimens with lower scores achieved the following 3 levels of accuracy in the remainder: 90% (Level 1), 95% (Level 2) and 98% (Level 3).

**Figure 2:**
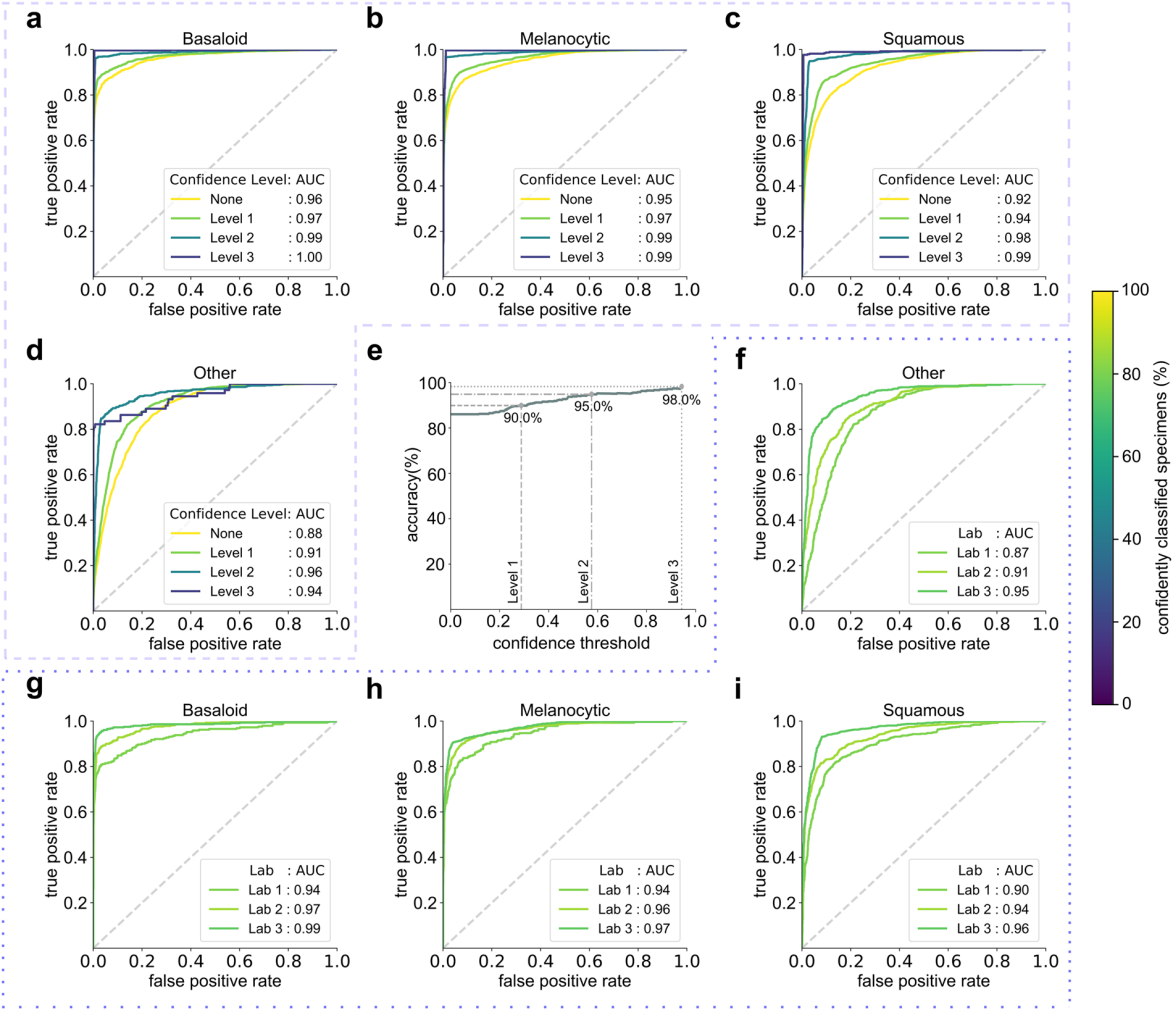
Receiver operating characteristic (ROC) curves are shown by lab, class, and confidence level for the test set of 13,537 images. ROC curves are shown for Basaloid (**a**,**g**), Melanocytic (**b**,**h**), Squamous (**c**,**i**) and Other (**d**,**f**) classes, with percentage of specimens classified for each curve represented by the colorbar at right. The three curves in each of (**a**–**d**) represent the respective thresholded confidence levels or no confidence threshold ("None”). The three curves in each of (**f**–**i**) represent the three labs. (**e**) Validation set accuracy in the Reference Lab is plotted versus sigmoid confidence score, with dashed lines corresponding to the sigmoid confidence thresholds set (and fixed) at 90% (Level 1), 95% (Level 2), and 98% (Level 3).

**Figure 3:**
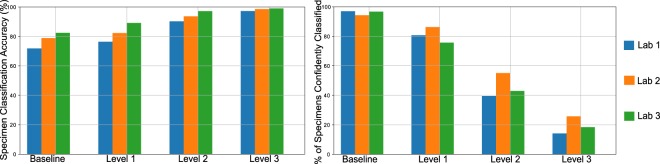
Test performance of the pathology deep learning system in each of the 3 test labs is illustrated. (left) Specimen classification accuracy is shown at each confidence level. (right) The percentage of specimens whose confidence score remains above the confidence threshold at each of the 3 confidence levels is shown. Note that even at baseline fewer than 100% of the specimens are classified. Some specimens are unclassified at baseline due to the lack of any ROI detected by CNN-2. This occurred in approximately 3–6% of specimens from each lab.

**Figure 4:**
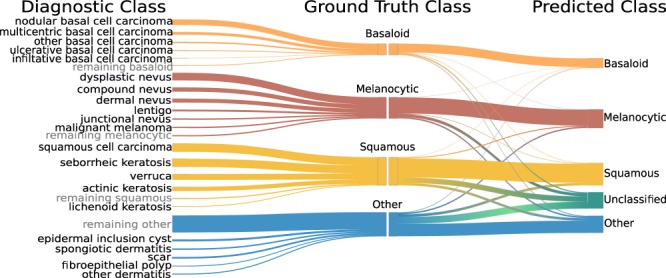
Sankey diagram depicting the mapping of ground truth classes to the top 5 most common diagnostic entities in the test set in each class (left). Malignant melanoma was not in the top 5 but included here due to its clinical importance. Also shown is the proportion of images correctly classified, along with the distribution of misclassifications and unclassified specimens (those for which confidence score was below the threshold) at confidence Level 1 (right). The width of each bar is proportional to the corresponding number of specimens found in the 3-lab test set.

To achieve high classification accuracy in the presence of a wide range of variability in tissue appearance between labs, a unique calibration set (about 520 WSIs) was collected from each lab and used to fine-tune the final classifier (CNN-3). Results are reported only on the test set, consisting of 13,537 WSIs from the 3 test labs which were not used in model training or development. The deep learning system effectively classifies WSIs into the 4 classes with an overall accuracy of 78% before thresholding on confidence score (referred to as baseline). Importantly, in specimens whose predictions exceeded the confidence threshold, the PDLS achieved an accuracy of 83%, 94%, and 98% for confidence levels 1, 2, and 3, respectively. Performance of the PDLS is characterized with receiver operating characteristic (ROC) curves, shown for each of the 4 classes in Fig. 2a–d at each confidence level; as confidence level increases, a larger percentage of images do not meet the threshold and are excluded from the analysis, as indicated by the colorbar. At Levels 1, 2, and 3, the percentage of total test specimens exceeding the confidence threshold was 81%, 46% and 20%, respectively. E.g., at Level 3, 20% of specimens still receive classification, while 80% are unclassified. Area under the curve (AUC) increased with increasing confidence level. Similar results are shown for Level 1 for each test lab in Fig. 2f–i which compare AUC and percentage of specimens confidently classified between the 3 labs. Figure 3 shows classification accuracy and the cumulative percentage of specimens which are classified at each confidence level and at baseline for the 3 test labs. Approximately 3–6% of specimens are not classified even at baseline due to the lack of any ROI detected by CNN-2. There is a trend towards lower inter-lab variability in accuracy and higher variability in percentage of specimens classified as confidence level increases. Figure 4 shows the mapping of ground truth class to the proportion correctly predicted as well as proportions confused for each of the other classes or remaining unclassified (at Level 1) due to lack of a confident prediction or absence of any ROI detected by CNN-2. Additionally, this figure shows the most common ground-truth diagnoses in each of the 4 classes found in the test set.

### Reduction of inter-site variance

**Figure 5:**
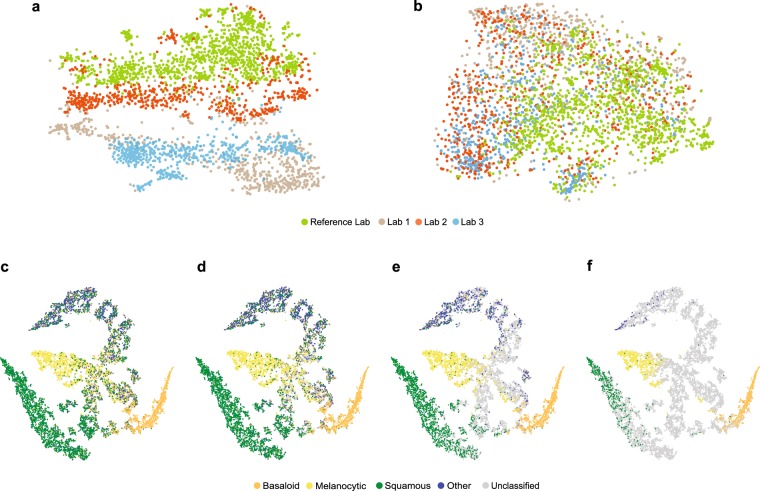
Image feature vectors are shown in 2-dimensional t-distributed stochastic neighbor embedded (t-SNE) plots. *Top:* Feature embeddings from CNN-2 are shown with (**a**) no prior image adaptation and (**b**) when image adaptation (using CNN-1) is performed prior to performing region of interest (ROI) extraction using CNN-2. Each point is an image patch within a whole slide image (WSI), colored by lab. *Bottom:* Feature embeddings from CNN-3, where each point represents a specimen and is colored according to ground-truth classification. (**c**) Specimens classified at baseline (no confidence threshold applied) are shown; (**d**–**f**) show increasing confidence thresholds (d = Level 1, e = Level 2, f = Level 3), with specimens not meeting the threshold in grey.

To demonstrate that the image adaptation performed by CNN-1 effectively reduces inter-site variations, we used t-distributed stochastic neighbour embedding (t-SNE) to compare the feature space of CNN-2 with and without first performing the image adaptation step. We show CNN-2’s embedded feature space *without* first performing image adaptation in Fig. 5a,b then shows the embedded feature space from CNN-2 when image adaptation is performed first. Inclusion of the image adaptation step results in more overlapped distributions in feature space than those produced without using image adaptation; this transformation into a common feature space allows the system to perform high-quality classification regardless of staining technique or scanner used.

### Effective class separation

Additionally, we used t-SNE to show class separation based on the internal feature representation learned by the final classifier (CNN-3), as shown in Fig. 5c. Each point in these t-SNE plots represents a single specimen with color denoting its ground-truth class. Figure 5d–f show the same information when thresholding at each of the 3 confidence levels (1–3, respectively), indicating in grey the specimens left unclassified at each. The clustering shows strong class separation between the 4 classes, with stronger separation and fewer specimens classified as confidence level increases.

### Timing profile

**Figure 6:**
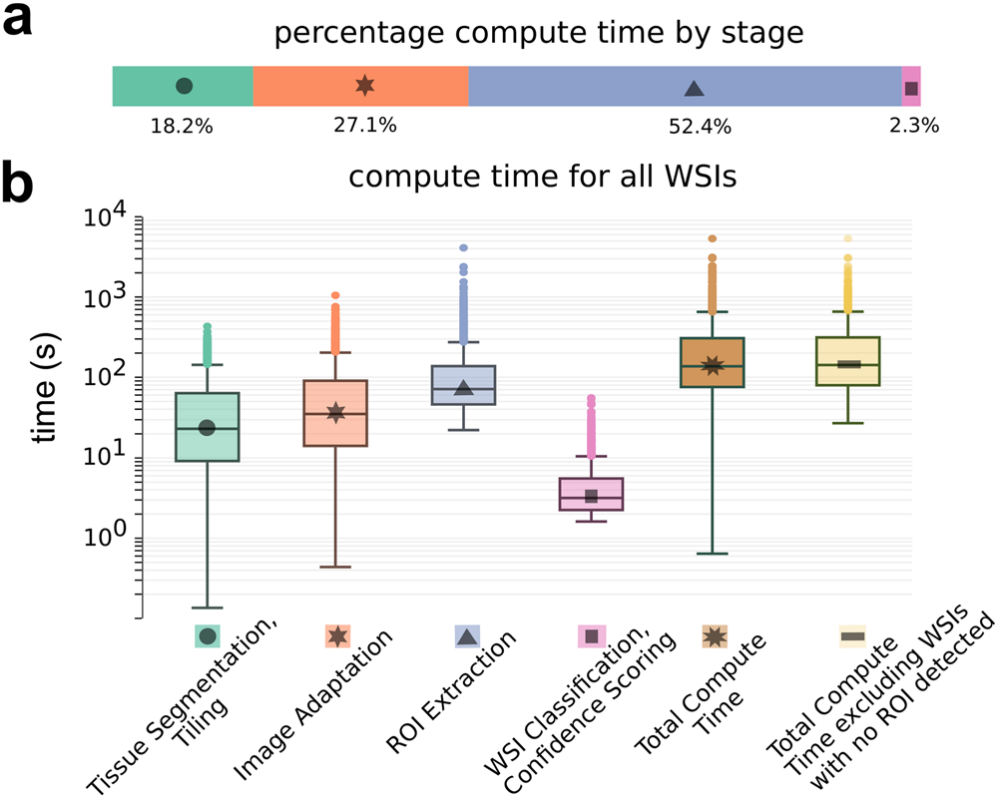
Compute time is shown for prediction by the pathology deep learning system (PDLS) on whole slide images from the calibration sets of the 3 test labs. (**a**) The median percentage of total computation time for each stage in the PDLS is shown. (**b**) A boxplot of the computation time in seconds required at each stage of the pipeline is shown on a logarithmic scale, along with total end-to-end execution time for all images (dark brown, median 137s), and excluding images for which no regions of interest are detected (light brown, median 142s).

It is important that execution time for any system intended to be implemented in a lab workflow be low enough to not present a bottleneck to diagnosis. Therefore, the proposed system was designed to be parallelizable across WSIs to enhance throughput and meet the efficiency demands of the real-world system. On a single compute node (described in Methods), the median processing time per WSI was 137 seconds, with overall throughput of 40 WSIs/hour. Fig. 6a shows the median time consumed by each stage in the pipeline, and Fig. 6b shows box-plots of time at each stage, as well as end-to-end execution time.

## Discussion

Our work demonstrates the ability of a multi-site generalizable PDLS to accurately classify the majority of specimens in a routine dermatopathology lab workflow. Developing a deep-learning-based classification which translates across image sets from multiple labs is non-trivial^25,26,30^. Without compensation for image variations, non-morphological differences between data from different labs are more prominent in the feature space than morphological differences between the specimens ultimately belonging to the same diagnostic classification. This is demonstrated in Fig. 5a, in which the image patches cluster according to the lab that prepared and scanned the corresponding slide. When image adaptation is performed prior to computing image features, the images do not strongly cluster by lab (Fig. 5b). Image adaptation (CNN-1) was found to be an important part of the PDLS, as neglecting this step can result in failure of CNN-2 to properly identify regions of interest. For example, when we eliminated the image adaptation step and attempted to directly predict on the test set from Lab 1, only 76% of specimens had *any* ROI detected, while including image adaptation allowed detection of an ROI in 98% of specimens. In this study, we demonstrate that a PDLS trained on a single Reference Lab can be effectively calibrated to 3 additional lab sites. Figures 5c–f show strong class separation between 4 classes, and this class separation strengthens with increasing confidence threshold. Intuitively, low-confidence images cluster at the intersection of the 4 classes. Strong class separation is reflected also in the ROC curves, which show high AUC across classes and labs, as seen in Fig. 2. AUC increases with increased confidence level, demonstrating the utility of confidence score thresholding as a tunable method for excluding poor model predictions. Figure 2d shows relatively worse performance in the Other class. In 5c it can be seen that there is some overlap between the Squamous and Other classes in feature space; Fig. 4 also shows some confusion between these two classes, but overall, demonstrates accurate classification of the majority of specimens from each class.

The majority of previous deep learning systems in digital pathology have been validated only on a single lab or scanner’s images^19,21^, curated datasets that ignored a portion of lab volume within a speciality^19,25,32^, and tested on small and unrepresentative datasets^19,21,32,35^, excluded images with artifacts^19,25,31^ or selectively reverse image "ground truth” retrospectively for misclassifications^25^ and train patch- or segmentation-based models while using traditional computer vision or heuristics to arrive at a whole slide prediction^19,29,31^. These methods do not lend themselves to real-world enabled deep learning systems that are capable of operating independent of the pathologist and prior to pathologist review. These systems would require some human intervention before they can provide useful information about a slide, and therefore do not enable improvements in lab workflow efficiencies.

In contrast, our PDLS is trained on *all* available slides– images with artifacts, slides without tissue on them, slides with poor staining or tissue preparation, slides exhibiting rare pathology, and those with very subtle evidence of pathology. All of this variability in the data necessitates that our PDLS is capable of determining when it is not likely to make a well-informed prediction. This is accomplished with a confidence score, which can be thresholded to obtain better system performance as shown in Fig. 2a–e. Correlation between system accuracy and confidence was established *a priori* using only the Reference Lab validation set (Fig. 2e) to fix the 3 confidence thresholds. By fixing thresholds *a priori* we establish that they are generalizable. Campanella *et al*.^25^ have attempted to similarly set a classification threshold which yields optimal performance; however, they perform this thresholding using the last layer output of a model, on the same test set in which they report it yielding 100% sensitivity; therefore they do not demonstrate the generalizability of this tuned parameter. Secondly, as Gal *et al.*^46^ demonstrate, a model’s predictive probability (last layer output) cannot be interpreted as a measure of confidence.

We report all performance measures (accuracy, AUC) at the level of a specimen, which may consist of several slides, since diagnosis is not reported at the slide level in dermatopathology. We aggregate all slide-level decisions to the specimen level as reported in Methods; this is particularly important as not all slides within a specimen will exhibit pathology, and therefore an incorrect prediction can be made if slide-level-reporting is performed. Similar systems^19,25,35,36^ have not attempted to solve the problem of aggregating slide-decisions to the specimen level at which diagnosis is performed.

For the PDLS to operate before pathologist assessment, the entire pipeline must be able to run in a time period that avoids delaying the presentation of a case to the pathologist. The compute time profile shown in Fig. 6a,b demonstrates that the PDLS can classify a WSI in under 3 minutes in the majority of cases, which is on the same order of the amount of time it takes for today’s scanners to scan a single slide. There was considerable variation in this number due to a large amount of variability in the size of the tissue. However, it is important to note that this process can be infinitely parallelized across WSIs to enhance throughput. Additional optimization of this process is possible and is the subject of future work.

There are several limitations to the current PDLS which are shared by previous implementations of deep learning image classification in digital pathology. First, when diagnosing a specimen, pathologists often have access to additional clinical information about the case, whereas our PDLS uses only WSIs to make a prediction. Training the PDLS with this additional clinical context as input would likely improve accuracy in some cases. A second limitation is that all existing systems for pathology classification attempt to put restrictions on the biology, namely that a WSI or a specimen can only represent a single diagnosis. However, in some specimens, multiple pathologic entities may be present, even within the same slide, which occasionally means that a single specimen can pertain to multiple classes. This occurrence is rare; rates of dual-class occurrence in each of the 3 test labs are provided in Supplementary Information (Table S1). We did not train the current PDLS to handle this special case since the available sample of images with dual ground-truth class is small; however, this will be a subject of future research.

While the current PDLS does not make diagnostic predictions, its classification has the potential to increase diagnostic efficiency and consistency. There are several scenarios for which the PDLS could provide such utility, though this requires further research in a prospective laboratory setting. For example, pathologists might choose to prioritize certain classes, e.g. Melanocytic, that may contain more difficult cases, requiring longer review time, additional levels ordered, or ancillary testing such as immunostains. Similarly, a dermatologist who interprets biopsies could choose to only receive cases classified as Basaloid, and avoid receiving many inflammatory cases or melanocytic lesions which might be sent for referral. The tunability of the confidence threshold in the model as a near-final step in assigning a classification has further implications for how this deep learning system might be utilized in practice. For example, for applications which can easily tolerate a small number of misclassifications (e.g. triage of cases to balance pathologists’ workloads), the desired confidence threshold could be lower, thereby avoiding an overly-large set of unclassified specimens. On the other hand, applications that involve identifying selected cases for secondary review (by a pathologist) might have a lower tolerance for misclassifications, but a higher tolerance for a large number of unclassified specimens; these applications might therefore make use of a high confidence threshold, prioritizing cases of discordance (between pathologist and PDLS); secondarily the threshold might be used for regulating the number of images subjected to re-review by rank-ordering cases based on the confidence score. Finally, as hierarchical classification models have been shown to outperform flat classifiers,^47^ we expect that the current PDLS serves as a basis for extension to diagnostic classification systems. This would enable further prioritization of more critical cases, such as those presenting features of melanoma.

## Conclusion

The techniques presented herein–namely deep learning of heterogeneously-composed classes, and confidence-based prediction screening– are not limited to application in dermatopathology or even pathology, but broadly demonstrate potentially effective strategies for translational application of deep learning in medical imaging. The PDLS presented delivers accurate prediction, regardless of scanner type or lab, and requires minimal calibration to achieve accurate results for a new lab. The system is capable of assessing which of its decisions are viable based on a computed confidence score, and thereby can filter out predictions that are unlikely to be correct. This confidence-based strategy is broadly applicable for achieving the low error rates necessary for the practical use of machine learning in challenging and nuanced domains of medical disciplines.

## Methods

### Data used in development

The proposed system was developed in its entirety using H&E-stained WSIs from Dermatopathology Laboratory of Central States, which is referred to as the Reference Lab in this work. All slides from this Reference Lab were scanned using the Leica Aperio AT2 Scanscope (Aperio, Leica Biosystems, Vista, California). This dataset is made up of two subsets, the first (3,070 WSIs) consisting of images representing commonly diagnosed dermatopathologic entities, and the second (2,000 slides) consisting of, in its entirety, an uninterrupted series of all cases sequentially accessioned over a period of less than a week, representing the typical distribution seen by the lab. This combined Reference Lab set of 5,070 WSIs was partitioned randomly into training (70%), validation (15%), and testing (15%) sets, such that WSIs from any given specimen were not split between sets.

### Taxonomy

The design of target classes in this study is heavily influenced by the prevalence of each class’s constituent pathologies and the presence of visually- and histologically-similar class-representative features. They capture, in roughly equal proportion, the majority of diagnostic entities seen in a dermatopathology lab practice. Specifically, we perform classification of WSIs into four classes: Basaloid, Squamous, Melanocytic, and Others. These four classes are defined by the following histological descriptions of their features:

1. *Basaloid*: Abnormal proliferations of basaloid-oval cells having scant cytoplasm and focal hyperchromasia of nuclei; cells in islands of variable size with round, broad-based and angular morphologies; peripheral palisading of nuclei, peritumoral clefting, and a fibromyxoid stroma.
2. *Squamous*: Squamoid epithelial proliferations ranging from a hyperplastic, papillomatous and thickened spinous layer to focal and full thickness atypia of the spinous zone as well as invasive strands of atypical epithelium extending into the dermis at various levels.
3. *Melanocytic*: Cells of melanocytic origin in the dermis, in symmetric, nested, and diffuse aggregates and within the intraepidermal compartment as single cell melanocytes and nests of melanocytes. Nests may be variable in size, irregularly spaced, and single cell melanocytes may be solitary, confluent, hyperchromatic, pagetoid and with pagetoid spread into the epidermis. Cellular atypia can range from none to striking anaplasia and may be in situ or invasive.
4. *Other*: Morphologic and histologic patterns that include either the absence of a specific abnormality or one of a wide variety of other neoplastic and inflammatory disorders which are both epithelial and dermal in location and etiology, and which do not belong to Classes 1-3.

These four classes account for more than 200 diagnostic entities in our test set, and their mapping to the most prevalent diagnostic entities in the test set is illustrated in Fig. 4.

### System design and training

Our image processing pipeline for the PDLS is illustrated in Fig. 1. The PDLS takes as input a WSI, segments out regions containing tissue, and divides these regions into a set of tiles, each of size 128 × 128 pixels, extracted at 10x magnification level. The process of assigning a label to a WSI using this set of tiles is comprised of three stages: (1) Image Adaptation, (2) Region of Interest Extraction, and (3) WSI Classification.

Since the PDLS is trained on only a single lab’s data, it is critical to perform image adaptation to adapt images received from test labs to a domain in which the image features are interpretable by the PDLS. Without adaptation, unaccounted-for variations in the images due to staining and scanning protocols can critically affect the performance of CNNs^25,26,30^. The PDLS performs image adaptation using a CNN (referred to as CNN-1), which is a 6-layer encoder-decoder network. Details of the architecture are shown in Supplementary Table S2. It takes as input an image tile and outputs an adapted tile of the same size and shape but with standardized image appearance. CNN-1 was trained using 300,000 tiles from the Reference Lab and mimics the average image appearance from the Reference Lab given an input tile.

Subsequently, ROI extraction is performed using a second CNN (referred to as CNN-2). This CNN is a segmentation network developed based on the U-Net architecture, described in Ronneberger *et al*.^48^ For training the network, a board-certified dermatopathologist annotated a subset of the training data from the Reference Lab for regions containing pathology. The proportion of Reference Lab datasets annotated from each class is included in Supplementary Table S3. During inference, CNN-2 takes input of a single tile and outputs a segmentation map. Tiles are selected corresponding to the positive regions of the segmentation map; the set of all identified tiles of interest, *t* from a WSI is passed on to the final stage classifier.

The final WSI classification is then performed using a third CNN (CNN-3). CNN-3 is a 10-layer WSI-level classifier trained end-to-end under the multiple instance learning (MIL) paradigm^49^. Details of its architecture can be found in Supplementary Table S4. Each tile *t* is treated as an instance of a bag which incorporates all of the extracted tiles within a given WSI. The output of this model is a 4 dimensional vector, each of whose elements represent the presence or absence of single class. The class corresponding to the maximum of this vector represents a label, *l* for the set of tiles *t* identified by CNN-2 where:

1 $$l\in \{{\rm{Basaloid}},\ {\rm{Squamous}},\ {\rm{Melanocytic}},\ {\rm{Others}}\}.$$

The convolution layers in all the CNNs use filters of size 3 × 3, and are followed by ReLU activation. All 3 CNNs are trained using cross-entropy loss. CNN-3 additionally outputs a confidence score for each WSI. In clinical practice, and in our dataset, diagnostic labels are reported at the level of a specimen, which may be represented by one or several WSIs. Therefore, the predictions of the PDLS are aggregated across WSIs to the specimen level; this is accomplished by assigning to a given specimen the maximum-confidence prediction across all WSIs representing that specimen.

### Calibration and validation for additional sites

To demonstrate its robustness to variations in scanners, staining, and image acquisition protocols, the PDLS was tested on 13,537 WSIs collected from 3 dermatopathology labs, representing two leading dermatopathology labs in top academic medical centers (Dermatopathology Center at Thomas Jefferson University and the Department of Dermatology at University of Florida College of Medicine) and a high volume national private dermatopathology laboratory (Cockerell Dermatopathology).We refer to these as *test labs*. Prior to the study, each lab sought study approval from the appropriate Institutional Review Board and was exempted. Each lab performed scanner validation prior to data collection, according to the guidelines of the College of American Pathologists^50^. Each test lab selected a date range within the past 4 years (based on slide availability) from which to scan, in its entirety, an uninterrupted series of sequentially accessioned cases comprising approximately 5,000 slides. The slides collected in each test lab are therefore equivalent to what would be collected in a prospective lab setting, in which the PDLS is installed in a lab immediately prior to collecting the first slide in our dataset, performs inference on each slide from each dermatopathology specimen accessioned in the lab from that date, and is uninstalled immediately following the last slide in our dataset. Each of the 3 test labs scanned their slides using a different scanner vendor and model at 20 × objective power. Scanner models used were: Leica Aperio AT2 Scanscope Console (Leica Biosystems, Vista, California), Hamamatsu Nanozoomer-XR (Hamamatsu Photonics, Hamamatsu City, Shizuoka, Japan), and 3DHistech Pannoramic 250 Flash III (3DHistech, Budapest, Hungary). The objective power and resolution of WSIs scanned at each site is reported in Table 1.

**Table 1:** Scanned objective power and resolution in microns per pixel is shown for each lab.

Lab	Resolution (microns/pixel)	Objective power
Reference Lab	0.25	40x
Lab 1	0.24	20x
Lab 2	0.50	20x
Lab 3	0.45	20x

**Figure 7:**
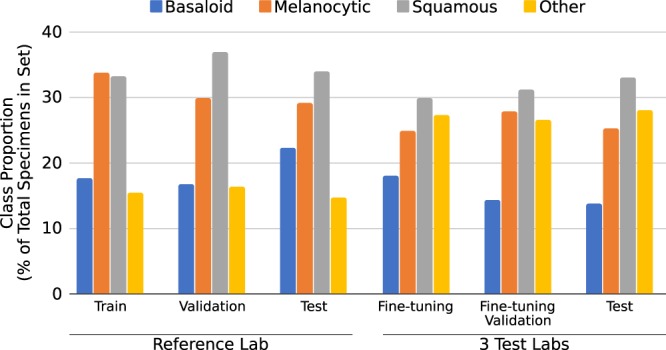
Ground truth relative class proportions are shown in terms of the percentage of total specimens in each set represented by each of the 4 classes (Basaloid, Melanocytic, Squamous and Other). Proportions are shown for the training, validation and test sets for the Reference lab (Left 3 groupings) and for the calibration set (fine-tuning and fine-tuning validation) and test set for all 3 test labs (Right 3 groupings).

All parameters and stages of the PDLS pipeline were held fixed after development on the Reference Lab, with the exception of CNN-3, whose weights were fine-tuned independently for each lab using a calibration set of approximately 520 WSIs. (We refer to this process as calibration). The calibration set for each lab consisted of approximately 500 sequentially-accessioned WSIs (pre-dating the test set) supplemented by 20 additional WSIs from melanoma specimens. Of these calibration images, 80% were used for fine-tuning, and 20% for lab-specific validation of the fine-tuning and image adaptation procedures. Specimens from the same patient were not split between fine-tuning, validation and test sets. After this calibration, all parameters were permanently held fixed, and the system was run only once on each lab’s test set of approximately 4,500 WSIs (range 4451 to 4585)– 13,537 in total. A comprehensive list of diagnoses found in each of the datasets included in this study is included as a Supplementary Dataset. Additionally, Fig. 7 shows the class balance for each set.

### Confidence scoring and threshold computation

Gal *et al*.^46^ propose a method to reliably measure the uncertainty of a decision made by a classifier. We have adapted this method for confidence scoring of the decision made by the PDLS. To determine a confidence score for a WSI we perform prediction on the same WSI repeatedly (using CNN-3) several times by omitting a random subset of neurons (here 70%) in CNN-3 from the prediction. Each repetition results in a prediction made using a different subset of feature representations. Here, we use *T* = 30 repetitions, where each repetition *i* yields a prediction *P*_*i*_, a vector of sigmoid values of length equal to the number of classes. Each element of *P*_*i*_ represents the binary probability, *p*_*i*,*c*_, of the corresponding WSI belonging to class *c*.

The confidence score *s* for a given WSI is then computed as follows:

2 $$s=ma{x}_{c}\left(\frac{\mathop{\sum }\limits_{i=1}^{T}{p}_{i,c}}{T}\right)$$

The class associated with the highest confidence *s* is the predicted class for the WSI. Finally, the specimen prediction is assigned as the maximum-confidence prediction of its constituent WSI predictions. If a specimen’s confidence score is below a certain threshold, then the prediction is considered unreliable and the specimen remains unclassified. Three threshold values for the confidence score were selected for analysis; these were determined during the development phase, using only the Reference lab’s data, because this confidence threshold is a parameter which can tune model performance. A single confidence threshold per level was computed and applied across all classes (i.e. there are no class-specific thresholds). Confidence thresholds were selected such that discarding specimens with sigmoid confidence lower than the threshold yielded a pre-defined level of accuracy in the remaining specimens of the validation set of the Reference Lab. The three target accuracy levels were 90%, 95% and 98%; the corresponding sigmoid confidence thresholds of 0.33, 0.76, and 0.99 correspond to confidence Levels 1, 2, and 3 respectively; these confidence thresholds were held fixed, and applied without modification to the test sets from the 3 test labs.

### Compute time

Compute time profiling of the PDLS was performed on an Amazon Web Services EC2 P3.8x large instance equipped with a 32-core Intel Xeon E5-2686 processor, 244GB RAM, and four 16GB NVIDIA Tesla V100 GPUs supported by NVLink for peer-to-peer GPU communication. Compute time was measured on the calibration sets of each of the the test labs.

## Supplementary information

Supplementary Information.

Supplementary Table.

## Data Availability

The datasets used in this study might be available upon reasonable request from the corresponding authors and with permission from the collaborating labs.

## Code availability

The PDLS is currently available as DermAI^TM^ from Proscia®. The source code is copyrighted by Proscia Inc. and is not available. No other custom code was used for this study.

## References

[1] Klipp, J. *The U.S. Anatomic Pathology Market: Forecast & Trends 2017–2020*. Laboratory Economics.

[2] Rogers HW, Weinstock MA, Feldman SR, Coldiron BM (2015). Incidence estimate of nonmelanoma skin cancer (keratinocyte carcinomas) in the US population, 2012. JAMA. Dermatol..

[3] Feramisco JD, Sadreyev RI, Murray ML, Grishin NV, Tsao H (2009). Phenotypic and genotypic analyses of genetic skin disease through the online mendelian inheritance in man (omim) database. J. Investig. Dermatol..

[4] Olhoffer IH, Lazova R, Leffell DJ (2002). Histopathologic misdiagnoses and their clinical consequences. Arch. Dermatol..

[5] Kent MN (2017). Diagnostic accuracy of virtual pathology vs traditional microscopy in a large dermatopathology study. JAMA Dermatol..

[6] Shah KK (2016). Validation of diagnostic accuracy with whole-slide imaging compared with glass slide review in dermatopathology. J. Am. Acad. of. Dermatol..

[7] Farmer ER, Gonin R, Hanna MP (1996). Discordance in the histopathologic diagnosis of melanoma and melanocytic nevi between expert pathologists. Hum. Pathol..

[8] Corona R (1996). Interobserver variability on the histopathologic diagnosis of cutaneous melanoma and other pigmented skin lesions. J. Clin. Oncol..

[9] Lodha S, Saggar S, Celebi JT, Silvers DN (2008). Discordance in the histopathologic diagnosis of difficult melanocytic neoplasms in the clinical setting. J. Cutan. Pathol..

[10] Elmore JG (2017). Pathologists’ diagnosis of invasive melanoma and melanocytic proliferations: observer accuracy and reproducibility study. BMJ.

[11] Shoo BA, Sagebiel RW, Kashani-Sabet M (2010). Discordance in the histopathologic diagnosis of melanoma at a melanoma referral center. J. Am. Acad. Dermatol..

[12] Baidoshvili A (2018). Evaluating the benefits of digital pathology implementation: time savings in laboratory logistics. Histopathology.

[13] Ho J (2014). Can digital pathology result in cost savings? a financial projection for digital pathology implementation at a large integrated health care organization. J Path Inform.

[14] Hanna MG (2019). Whole slide imaging equivalency and efficiency study: experience at a large academic center. Mod. Pathol..

[15] Al-Janabi S, Huisman A, Van Diest PJ (2012). Digital pathology: current status and future perspectives. Histopathology.

[16] Cruz-Roa A (2017). Accurate and reproducible invasive breast cancer detection in whole-slide images: A deep learning approach for quantifying tumor extent. Sci. Rep..

[17] Litjens G (2016). Deep learning as a tool for increased accuracy and efficiency of histopathological diagnosis. Sci. Rep..

[18] Ardila D (2019). End-to-end lung cancer screening with three-dimensional deep learning on low-dose chest computed tomography. Nat. Med..

[19] Olsen TG (2018). Diagnostic performance of deep learning algorithms applied to three common diagnoses in dermatopathology. J. Pathol. Inform..

[20] Esteva A (2017). Dermatologist-level classification of skin cancer with deep neural networks. Nature.

[21] Li, J. *et al*. An attention-based multi-resolution model for prostate whole slide image classification and localization. CVPR Workshop Towards Causal Explainable & Universal MVD (2019).

[22] Abràmoff MD (2018). Pivotal trial of an autonomous AI-based diagnostic system for detection of diabetic retinopathy in primary care offices. NPJ Digit. Med..

[23] Yao, L. *et al*. Learning to diagnose from scratch by exploiting dependencies among labels. Preprint at https://arxiv.org/abs/1710.10501 (2017).

[24] Hwang Eui Jin, Park Sunggyun, Jin Kwang-Nam, Kim Jung Im, Choi So Young, Lee Jong Hyuk, Goo Jin Mo, Aum Jaehong, Yim Jae-Joon, Cohen Julien G., Ferretti Gilbert R., Park Chang Min (2019). Development and Validation of a Deep Learning–Based Automated Detection Algorithm for Major Thoracic Diseases on Chest Radiographs. JAMA Network Open.

[25] Campanella G (2019). Clinical-grade computational pathology using weakly supervised deep learning on whole slide images. Nat. Med..

[26] Tellez David, Litjens Geert, Bándi Péter, Bulten Wouter, Bokhorst John-Melle, Ciompi Francesco, van der Laak Jeroen (2019). Quantifying the effects of data augmentation and stain color normalization in convolutional neural networks for computational pathology. Medical Image Analysis.

[27] Korbar B (2017). Deep learning for classification of colorectal polyps on whole-slide images. J. Path. Inform..

[28] Sornapudi S (2018). Deep learning nuclei detection in digitized histology images by superpixels. J. Path. Inform..

[29] Awan, R., Koohbanani, N. A., Shaban, M. & Rajpoot, N. Context-aware learning using transferable features for classification of breast cancer histology images. *Int. Conf. Image Anal. & Recog.* 788–795 (2018).

[30] Ciompi, F. *et al*. The importance of stain normalization in colorectal tissue classification with convolutional networks. *Proc IEEE Int Sym Biomed Imaging* 160–163, 10.1109/ISBI.2017.7950492 (2019).

[31] Bulten, W. *et al*. Automated deep-learning system for Gleason grading of prostate cancer using biopsies: a diagnostic study. *Lancet Oncol*. (2020). 10.1016/S1470-2045(19)30739-9 31926805

[32] Ghaznavi F, Evans A, Madabhushi A, Feldman M (2013). Digital imaging in pathology: Whole-slide imaging and beyond. Annu Rev Pathol-Mech.

[33] Bejnordi BE (2018). Using deep convolutional neural networks to identify and classify tumor-associated stroma in diagnostic breast biopsies. Mod. Pathol..

[34] Janowczyk A, Madabhushi A (2016). Deep learning for digital pathology image analysis: A comprehensive tutorial with selected use cases. J. Path. Inform..

[35] Hart SN, Flotte W, Andrew P (2019). Classification of melanocytic lesions in selected and whole - slide images via convolutional neural networks. J. Pathol. Inform..

[36] Ing, N. *et al*. A deep multiple instance model to predict prostate cancer metastasis from nuclear morphology. In *Proc Int Conf Med Imag Deep Learning* (2018).

[37] Kohlberger Timo, Liu Yun, Moran Melissa, Chen Po-HsuanCameron, Brown Trissia, Hipp JasonD, Mermel CraigH, Stumpe MartinC (2019). Whole-slide image focus quality: Automatic assessment and impact on ai cancer detection. Journal of Pathology Informatics.

[38] Senaras, C., Niazi, M. K. K., Lozanski, G. & Gurcan, M. N. DeepFocus: detection of out-of-focus regions in whole slide digital images using deep learning. *PLoS ONE* **13** (2018). 10.1371/journal.pone.0205387 PMC6201886 30359393

[39] Janowczyk, A., Zuo, R., Gilmore, H., Feldman, M. & Madabhushi, A. HistoQC: an open-source quality control tool for digital pathology slides. *JCO. Clin. Cancer. Inform*. 1–7, 10.1200/CCI.18.00157 (2019). 10.1200/CCI.18.00157 PMC6552675 30990737

[40] Bostwick DG, Liu L, Brawer MK, Qian J (2012). High-grade prostatic intraepithelial neoplasia. Korean. J. Urol..

[41] Zhou M (2018). High-grade prostatic intraepithelial neoplasia, PIN-like carcinoma, ductal carcinoma, and intraductal carcinoma of the prostate. Mod. Pathol..

[42] Zynger DL, Yang X (2009). High-grade prostatic intraepithelial neoplasia of the prostate: The precursor lesion of prostate cancer. Int. J. Clin. Exp. Pathol..

[43] Ali, S. & May, C. V. Ink removal from histopathology whole slide images by combining classification, detection and image generation models. *ISBI.* 928–932 (2019).

[44] Ström, P. *et al*. Pathologist-level grading of prostate biopsies with artificial intelligence. Preprint at http://arxiv.org/abs/1907.01368 (2019).

[45] Oakden-Rayner, L., Dunnmon, J., Carneiro, G. & Ré, C. Hidden stratification causes clinically meaningful failures in machine learning for medical imaging. Preprint at http://arxiv.org/abs/1909.12475 (2019). 10.1145/3368555.3384468 PMC7665161 33196064

[46] Gal, Y. & Ghahramani, Z. Dropout as a Bayesian approximation: Representing model uncertainty in deep learning. In *Int. Conf. on. Machine. Learning*., 1050–1059 (2016).

[47] Silva-Palacios D, Ferri C, Ramírez-Quintana MJ (2018). Probabilistic class hierarchies for multiclass classification. J. Comput. Sci..

[48] Ronneberger, O., Fischer, P. & Brox, T. U-Net: Convolutional networks for biomedical image segmentation. In *Med. Image. Comput. Comput. Assist. Interv*., 234–241 (Springer, 2015).

[49] Dietterich TG, Lathrop RH, Lozano-Pérez T (1997). Solving the multiple instance problem with axis-parallel rectangles. Artif. Intell..

[50] Pantanowitz L (2013). Validating Whole Slide Imaging for Diagnostic Purposes in Pathology. Arch. Pathol. Lab. Med..

