# Supplementary material for: Tailored for Real-World: A Whole Slide Image Classification System Validated on Uncurated Multi-Site Data Emulating the Prospective Pathology Workload
Source: Sci Rep. 2020 Feb 21;10:3217. doi: 10.1038/s41598-020-59985-2 (PMC7035316; doi:10.1038/s41598-020-59985-2)
Supplement: Supplementary file 1 — Supplementary Information. [file 41598_2020_59985_MOESM1_ESM.pdf]

Tailored for Real-World: A Whole Slide Image Classification System Validated on Uncurated Multi-Site Data Emulating the Prospective Pathology Workload

Julianna D. Ianni[1, *, †], Rajath E. Soans[1, *, †], Sivaramakrishnan Sankarapandian[1], Ramachandra Vikas Chamarthi[1], Devi Ayyagari[1], Thomas G. Olsen[2, 3], Michael J. Bonham[1], Coleman C. Stavish[1], Kiran Motaparthi[4], Clay J. Cockerell[5], Theresa A. Feeser[1], and Jason B. Lee[6]

[1]Proscia Inc., Philadelphia, Pennsylvania, USA.
[2]Department of Dermatology, Boonshoft School of Medicine, Wright State University School of Medicine, Dayton, Ohio, USA
[3]Dermatopathology Laboratory of Central States, Dayton, Ohio, USA
[4]Department of Dermatology, University of Florida College of Medicine, Gainesville, Florida
[5]Cockerell Dermatopathology, Dallas, Texas, USA.
[6]Departments of Dermatology and Cutaneous Biology, Sidney Kimmel Medical College at Thomas Jefferson University, Philadelphia, Pennsylvania, USA.
[*]julianna@proscia.com, rajath@proscia.com
[†]These authors contributed equally to this work.

SUPPLEMENTARY INFORMATION

Lab	specimens (%)	
	two classes	three classes
Lab 1	2.26	0.04
Lab 2	1.35	0
Lab 3	2.83	0

Table S1. Shown is the percentage of specimens from each of the 3 test labs which exhibit multiple pathologic entities corresponding to more than one of the 4 classes. This applies to fewer than 3% of specimens in each lab. The Pathology Deep Learning System (PDLS) was not designed to predict multiple classes for a single specimen, though this is a subject for future research.

Layer No.	Layer type [(size)/stride, pad]
1	Conv [(3 x 3) / 2, 1] + ReLU
2	Conv [(3 x 3) / 2, 1] + ReLU
3	Conv [(3 x 3) / 2, 1] + ReLU
4	Conv Transpose [(3 x 3) / 2, 1] + ReLU
5	Conv Transpose [(3 x 3) / 2, 1] + ReLU
6	Conv Transpose [(3 x 3) / 2 ,1] + ReLU

Table S2. The architecture of CNN-1 is shown. CNN-1 is used in image adaptation step; The first three convolution layers employ 3×3 kernels with stride of 2 pixels to generate an encoded vector of the input. Using this encoded vector, the last three Transposed-Convolution layers reconstruct the output with image appearance adapted to Reference Lab.

Class	Percentage of Reference Lab WSIs annotated in training set	validation set	test set
Basaloid	3.75	4.18	4.26
Melanocytic	4.84	4.32	4.38
Squamous	3.99	5.16	3.87
Total annotated	12.58	13.66	12.51

Table S3. A subset of whole slide images (WSIs) from the Reference Lab was selected for annotation by a board-certified dermatopathologist for use in training, validating and testing the ROI extraction model (CNN-2). This set was comprised of samples from each of the primary target classes (Basaloid, Squamous, and Melanocytic). Shown are the proportions of the Reference Lab training, validation, and test sets which received annotations.

Layer No.	Layer name [(size)/stride, pad]
1	Conv [(3 x 3) / 1, 1] + ReLU
2	Conv [(3 x 3) / 1, 1] + ReLU
3	Conv [(3 x 3) / 1, 1] + ReLU
4	Conv [(3 x 3) / 1, 1] + ReLU
5	Conv [(3 x 3) / 1, 1] + ReLU
6	FC [2048] + ReLU
7	FC [1024] + ReLU
8	MIL Pooling
9	FC [512] + ReLU
10	FC [4]

Table S4. The architecture of CNN-3 is shown. In the final stage of the Pathology Deep Learning System (PDLS), the classifier is trained to perform a four-way classification with input tiles from a whole slide image corresponding to regions of interest identified by CNN-2. CNN-3 is comprised of five convolution layers followed by two Fully-Connected layers; this results in a feature vector for the input bag which is fed to layer 8 which is an multiple instance pooling layer. Layers 9 and 10 then use the layer 8 output to classify the input bag into the 4 classes.
